# Plant–Herbivore–*Trichogramma* Interactions: Chemical Ecology, Host Quality, Diapause Regulation, and Implications for Biological Control

**DOI:** 10.3390/plants15213330

**Published:** 2026-10-28

**Authors:** Chen Zhang, Lin Zhu, Nan Wang, Zhi-Da Li, Yu Wang

**Affiliations:** 1 Agricultural College, Jilin Agricultural Science and Technology College, Jilin 132101, China; zhangchenjl@163.com (C.Z.); wangnan0488@163.com (N.W.); lzz1023080787@gmail.com (Z.-D.L.); 2 School of Civil Engineering and Transportation, Changchun University of Architecture and Civil Engineering, Changchun 130607, China; zhulin202608@163.com

**Keywords:** *Trichogramma*, diapause, plant–herbivore–parasitoid interactions, tritrophic interactions, plant volatiles, host quality, biological control, climate change

## Abstract

Diapause is an important seasonal adaptation that enables *Trichogramma* egg parasitoids to survive unfavorable environmental conditions and maintain synchrony with host availability. Although temperature and photoperiod are established regulators of diapause, plants can influence parasitoid ecology through changes in host availability, volatile emissions, defense responses, host egg quality, and local environmental conditions. This review synthesizes evidence on plant–herbivore–*Trichogramma* interactions and examines their potential relevance to diapause regulation and biological control. Studies demonstrate that plant species, host-plant phenology, herbivore-induced and oviposition-induced volatiles, and plant defense responses can affect parasitoid host searching and parasitism. Plant-mediated changes in host nutrition and plant architecture also influence parasitoid development, dispersal, and performance, although responses vary among species and environmental conditions. We further review evidence on the regulation of *Trichogramma* diapause by temperature, photoperiod, maternal effects, metabolic pathways, and population-specific responses, together with recent findings on lipid metabolism, carbohydrate transport, endocrine signaling, and symbiont-associated variation. Importantly, direct experimental evidence linking plant-derived cues to diapause induction remains limited. Climate change may further affect parasitoid–host synchrony by altering thermal conditions, plant development, host phenology, and extreme temperature exposure. Future research should integrate plant chemistry, host egg quality, maternal conditions, and physiological measurements with direct assessments of diapause under realistic environmental conditions. Such studies may improve our understanding of seasonal adaptation and support more reliable *Trichogramma*-based biological control.

## Simple Summary

Plants influence the survival and performance of *Trichogramma* egg parasitoids by affecting host availability, chemical signals, host egg quality, and environmental conditions. These parasitoids also undergo diapause, a developmental arrest that helps them survive unfavorable seasons. Temperature and photoperiod are major regulators of diapause, but plants may indirectly influence seasonal adaptation through their effects on herbivores and parasitoid habitats. This review examines plant–herbivore–*Trichogramma* interactions, environmental and physiological regulation of diapause, and the potential effects of climate change. Current studies show that plant-derived signals influence parasitoid host searching and biological-control performance, but their direct role in diapause induction remains unclear. Future research should investigate whether plant-associated conditions influence maternal responses, offspring diapause, and seasonal synchrony. A better understanding of these relationships may help improve the reliability of *Trichogramma*-based biological control under changing environmental conditions.

## 1. Introduction

*Trichogramma* species are widely used as egg parasitoids in agricultural biological control because they parasitize the eggs of economically important herbivorous pests [1,2,3]. Their field performance depends on host egg availability and suitability, the ability to locate suitable hosts, and environmental conditions influencing parasitoid survival and reproduction [4,5]. Plants influence these processes by shaping herbivore oviposition patterns, providing chemical cues for host location, and affecting parasitoid searching efficiency through differences in plant architecture and surface characteristics [6]. In addition to these ecological determinants of field performance, diapause represents an important seasonal adaptation that enables insects to survive predictable periods of environmental adversity through regulated developmental arrest and associated metabolic adjustments [7,8,9,10,11]. Distinguishing diapause from other forms of developmental delay requires evidence of regulated developmental programming rather than delayed development alone. Although temperature and photoperiod are major regulators of diapause [8,12,13], plant- and herbivore-derived cues provide information relevant to host location and habitat suitability [14,15,16]. Whether these cues also influence diapause induction in *Trichogramma* remains to be established experimentally.

Plants function not only as physical environments but also as information sources within multitrophic systems. Through herbivore-induced plant volatiles (HIPVs), oviposition-induced plant volatiles (OIPVs), and defense-related chemical responses, plants provide parasitoids with indirect information about host presence and habitat suitability [17,18,19]. Such chemical communication influences parasitoid searching behavior, parasitism efficiency, and ecological interactions across trophic levels [18,20]. However, whether plant-derived information directly or indirectly influences diapause induction in *Trichogramma* remains experimentally unresolved. Plant condition may influence parasitoid seasonal adaptation through several interconnected pathways. Plant phenology determines herbivore abundance and egg availability, while plant nutritional status influences herbivore quality and consequently parasitoid performance. In addition, plant-associated microclimates can modify temperature, humidity, and light conditions experienced by developing parasitoids. These interactions suggest that diapause regulation should be considered within a broader tritrophic framework rather than as an isolated parasitoid trait [12,21,22,23,24]. Recent advances in insect physiology have further demonstrated that diapause is controlled by integrated molecular networks involving endocrine signaling, insulin/TOR pathways, lipid metabolism, carbohydrate regulation, and stress-response mechanisms [11,25]. In *Trichogramma*, transcriptomic and functional studies have revealed extensive metabolic restructuring during diapause, highlighting the importance of energy storage and physiological preparation before developmental arrest.

Climate change can alter temperature patterns, precipitation, plant development, herbivore phenology, and the frequency of extreme events [26,27,28]. These changes may influence host availability, parasitoid performance, and diapause timing through different ecological and physiological pathways. This review synthesizes (i) experimentally supported plant-mediated influences on *Trichogramma* ecology and biological control, (ii) environmental and biological factors regulating diapause, (iii) molecular and physiological mechanisms underlying diapause, and (iv) the potential consequences of climate variability for parasitoid–host synchrony.

## 2. Plant–Herbivore–*Trichogramma* Interactions: Ecological and Biological Control Implications

### 2.1. Host-Plant Phenology and Spatial and Temporal Availability of Host Eggs

Host-plant phenology can influence the spatial and temporal availability of herbivore eggs to *Trichogramma* by altering plant suitability for herbivore oviposition. This relationship is evident in *Helicoverpa armigera*, which lays substantially more eggs on reproductive-stage pigeonpea than on vegetative plants [29]. Similarly, flowering tobacco and sunflower receive greater oviposition by *H. armigera* than non-flowering plants [30]. More broadly, herbivorous insects often adjust oviposition in response to plant age and condition. Reviewing 71 studies involving 62 herbivorous insect species, Jones [31] found that females generally preferred plants that provided more favorable conditions for offspring development, particularly on native host plants. These findings indicate that plant developmental stage and physiological condition can influence herbivore oviposition preferences and, consequently, the spatial distribution of host eggs among available plants. Although seasonal changes in plant phenology may also affect the timing of host egg availability, the cited oviposition-preference studies do not establish that herbivores delay egg laying. Therefore, spatial redistribution of oviposition and temporal changes in egg availability should be considered separately when evaluating host availability to *Trichogramma*.

For egg parasitoids, changes in host-plant phenology can influence both the duration of host availability and the conditions under which hosts are encountered. In maize, Kuhar et al. [32] reported that *Trichogramma ostriniae* parasitized *Ostrinia nubilalis* eggs during the host oviposition period. Although this observation illustrates the importance of host egg availability for parasitoid activity, the study does not independently establish an effect of crop phenology on parasitism or diapause. Plant development can further modify parasitoid foraging conditions, as plant size, architecture, egg-mass distribution, and weather conditions influence the probability of host discovery and parasitism [33]. Plant ontogeny also alters the chemical environment used by natural enemies during host searching. In *Datura wrightii,* Hare [34] found that herbivore-induced volatile production was greatest during vegetative growth, declined after flowering and fruiting began, and was no longer inducible by September. These findings indicate that crop development changes both the availability of host eggs and the physical and chemical conditions under which *Trichogramma* searches for them. Host abundance, egg distribution, plant architecture, and the timing of parasitoid activity together determine opportunities for parasitism.

### 2.2. Effects of Plant Species, Cultivars, and Genotypes on Trichogramma Performance

Plant identity can strongly influence how *Trichogramma* exploit plant-derived information, but these responses also depend on parasitoid genotype. Afentoulis et al. [35] showed that attraction of *Trichogramma evanescens* to oviposition-induced volatiles differed among *Brassica* species and parasitoid lines. The DG018 line was attracted to egg-infested *Brassica nigra* 72 h after oviposition, whereas the Bawc line responded to *Brassica napus* after 24 h; the BB line did not respond significantly to any tested species. In *Brassica rapa*, 65% of DG018 females preferred volatiles from egg-infested plants expressing hypersensitive response (HR)-like necrosis. These results demonstrate that the value of plant-derived cues depends on the specific plant–parasitoid combination rather than representing a uniform response across crops or parasitoid populations. Plant-derived cues also vary in their attractiveness depending on crop species and the time elapsed after herbivore activity. Sousa et al. [36] found that *Trichogramma pretiosum* was more attracted to rice and maize plants 24 h after *Spodoptera frugiperda* herbivory than to undamaged plants. Oviposited rice plants were also preferred over controls, whereas oviposited maize became more attractive after 48 h. Moreover, parasitism of sentinel eggs increased near herbivore-damaged plants. Together, these findings indicate that herbivore-associated changes in plant volatile emissions can influence *Trichogramma* host-searching behavior, with responses varying across crop species and experimental conditions.

Plant effects can also operate indirectly through host quality. Moreau et al. [37] showed that grapevine cultivar influenced the quality of *Lobesia botrana* eggs subsequently encountered by *T. evanescens*: cultivars associated with larger host eggs generally supported greater parasitism, improved parasitoid survival, and faster development. However, plant resistance does not always improve the compatibility between resistant crops and biological control agents. Zannou et al. [38] found that the resistant tomato cultivar Corona F1 and *Solanum neorickii* did not substantially impair parasitoid performance, whereas highly resistant *Solanum arcanum*, characterized by dense glandular trichomes, reduced the performance of the tested natural enemies, including *Trichogramma achaeae*. In addition, Nascimento et al. [39] investigated the olfactory responses of *T. pretiosum* to herbivore-induced volatiles from Bt and non-Bt maize plants using laboratory Y-tube olfactometer assays with intact, greenhouse-grown plants as odor sources. Females showed stronger attraction to volatiles induced by nocturnal *Spodoptera frugiperda* feeding in non-Bt maize than to those from Bt hybrids, indicating that plant genotype and herbivory timing can influence parasitoid olfactory responses.

The surrounding arthropod community can further modify these plant-mediated effects. Cascone et al. [40] demonstrated that feeding by the zoophytophagous predator *Nesidiocoris tenuis* significantly altered tomato volatile profiles and increased attraction of *T. achaeae*, both with and without *Tuta absoluta* infestation. Treatments involving *N. tenuis* also significantly increased total volatile emissions, while combinations with *T. absoluta* altered the relative abundance of compounds such as β-pinene, myrcene, and methyl salicylate. These findings show that the chemical environment encountered by *Trichogramma* reflects interactions among the plant, host, parasitoid, and other arthropods rather than plant genotype alone. Together, these studies show that plant species, cultivar, and genotype can modify parasitoid attraction, host egg suitability, and biological control outcomes. Effects are not uniform: resistant plants may facilitate, have little effect on, or interfere with parasitoid performance depending on their chemical and physical traits. Consequently, plant resistance and compatibility with *Trichogramma* should be evaluated jointly using host oviposition, parasitoid orientation, parasitism, development, and emergence rather than inferred from resistance traits alone.

### 2.3. Herbivore-Induced Plant Volatiles and Host Location

Herbivore-induced plant volatiles (HIPVs) provide an important indirect mechanism through which plants influence *Trichogramma* host location [41]. Herbivore feeding alters plant metabolism and volatile emissions, producing odor blends that parasitoids can use to locate habitats where hosts are likely to occur [42]. These cues are particularly useful to egg parasitoids because host eggs are small and difficult to detect from a distance [43]. Plant-derived odors may help parasitoids locate host-associated patches, while more specific host-associated cues assist in detecting and assessing individual eggs. Ali and Wright [44] found that *Trichogramma papilionis* responded more strongly when host eggs were presented with plant material than when either was presented alone. These findings suggest that plant- and host-associated cues can act together during host searching.

However, herbivore-induced plant volatiles do not always increase parasitoid attraction. Gontijo et al. [45] found that *T. achaeae* was innately attracted to tomato odors and responded to the sex pheromone of *Tuta absoluta*, but did not consistently distinguish between odors from uninfested and *T. absoluta*-infested tomato plants. These findings indicate that herbivore-induced changes in plant volatiles do not necessarily enhance parasitoid attraction. The effectiveness of HIPVs may therefore depend on the plant–herbivore combination, volatile composition, and parasitoid sensory responses.

Previous experience can also influence how *Trichogramma* responds to plant odors. Gonthier et al. [46] demonstrated that previous exposure to tomato odors altered searching behavior in *T. achaeae*, *T. evanescens*, and *Trichogramma dendrolimi*, although effective conditioning procedures differed among species. Associative experience increased searching responses in *T. achaeae* and *T. evanescens*, but increased parasitism only in *T. achaeae*. These findings indicate that the effects of previous experience on host searching and parasitism differ among *Trichogramma* species. Together, these studies show that plant-derived volatiles influence *Trichogramma* host searching, with responses depending on volatile composition, parasitoid species, and previous experience. Future studies should combine volatile analysis with behavioral assays and field measurements of host discovery and parasitism to determine which chemical cues consistently improve biological-control performance.

### 2.4. Oviposition-Induced Plant Volatiles (OIPVs) and Host Egg Detection

Oviposition-induced plant volatiles (OIPVs) can help *Trichogramma* locate host eggs before herbivore larvae begin feeding. During oviposition, insects may deposit substances that trigger changes in plant volatile emissions. Pashalidou et al. [47] showed that *T. evanescens* responded to Brussels sprout cues induced by *Pieris rapae* oviposition. Female accessory reproductive gland secretions containing the anti-aphrodisiac compound indole also induced plant cues attractive to the parasitoids. Previous associative experience enabled parasitoids to respond to plant cues induced by indole alone, indicating that learning can influence their responses to oviposition-related odors.

Parasitoid responses to OIPVs also vary with time after oviposition and among species. Milonas et al. [48] found that *Tuta absoluta* oviposition caused qualitative and quantitative changes in tomato volatile emissions, with 68 compounds detected across treatments and a significant increase in total volatile emissions. Antennae of both tested *Trichogramma* species detected several compounds, but only *Trichogramma cordubensis* showed significant attraction to oviposited plants, specifically 24 h after egg deposition. Attraction was absent at 48 and 72 h, while *T. achaeae* showed no significant preference at any tested interval. These findings indicate that detecting OIPVs does not always result in behavioral attraction and that parasitoid responses can change with time after oviposition.

Plant volatiles may also provide information about host egg quality. Tian et al. [49] found that *Trichogramma japonicum* preferred odors from rice plants bearing two-day-old *Cnaphalocrocis medinalis* eggs over plants bearing younger or older eggs. Two-day-old eggs also showed higher parasitism, shorter parasitoid development, and greater offspring emergence. Chemical analysis identified increased emissions of D-limonene and α-pinene from plants bearing two-day-old eggs. Synthetic versions of these compounds attracted the parasitoids, whereas rice plants genetically deficient in their production lost this attractiveness. Restoring these compounds restored parasitoid attraction. These findings suggest that OIPVs can help parasitoids locate host eggs associated with better developmental outcomes.

In addition to plant volatiles, *Trichogramma* can respond to chemical cues released directly from host eggs. Kong et al. [50] identified 13 compounds specific to *Ostrinia furnacalis* egg volatiles, of which six—oleic acid, stearic acid, dioctyl phthalate, tetracosane, palmitic acid, and 4-hydroxyphenylethanol—were attractive to female *T. ostriniae*. A synthetic blend of these compounds attracted females across several concentrations, and cage and field experiments showed higher parasitism of *O. furnacalis* eggs treated with the attractive blend than untreated controls. Combining the egg-volatile blend with host sex-pheromone components further improved parasitism. These findings suggest that plant-derived and egg-derived chemical cues may provide complementary information during parasitoid host searching.

Studies of chemosensory genes and proteins have also improved our understanding of how *Trichogramma* detects host-associated chemical cues. Transcriptomic analysis of *T. japonicum* identified 66 chemosensory receptor genes, including 51 odorant receptors, with 65 of the 66 receptors highly expressed in the head and greater representation of chemosensory-related genes in females than males [51]. Similarly, *T. pretiosum* possesses at least 22 odorant-binding proteins and 105 odorant receptors [52]. More importantly, Kong et al. [53] provided functional evidence in *T. ostriniae*: TostOBP12 was the most highly expressed odorant-binding protein in female heads and bound both host sex-pheromone components and egg-surface compounds. Silencing TostOBP12 by RNA interference abolished the behavioral response to the host sex pheromone without affecting parasitoid development. These findings identify TostOBP12 as an important protein involved in responses to host-associated chemical cues. Together, these studies show that *Trichogramma* uses plant- and host-associated chemical cues during host searching, with responses varying according to volatile composition, time since oviposition, and parasitoid species. Future studies should determine whether identified attractive compounds consistently improve host location and parasitism under field conditions.

### 2.5. Plant Defense Responses and Their Implications for Biological Control

Plant defense activation can influence *Trichogramma*-based biological control by affecting herbivore performance and the volatile cues used by parasitoids during host searching. These effects may be beneficial when induced resistance suppresses herbivores while enhancing parasitoid attraction, but they may also reduce parasitoid recruitment if the resulting volatile profile becomes less attractive. Weber et al. [54] found that methyl jasmonate applied directly to host eggs did not affect *T. pretiosum* chemotaxis or parasitism, whereas treatment of tomato plants increased parasitoid foraging and parasitism. These findings indicate that the observed effects were associated with plant-mediated changes rather than direct exposure of parasitoids to methyl jasmonate. Similarly, Rani and Murali-Baskaran [55] reported that jasmonic-acid treatment in wheat reduced pink stem borer dead-heart and white-ear symptoms by 31.7% and 27.9%, respectively, while increasing grain yield by 28.9%. Volatile analysis identified compounds, including octadecane and eicosane, associated with enhanced foraging responses of *Trichogramma chilonis*. These studies suggest that induced plant defense can improve crop protection while supporting parasitoid activity.

However, defense activation does not always enhance parasitoid recruitment. Zhu et al. [56] demonstrated that treatment of rice with the plant immunity inducer Atailing (provided by the Institute of Plant Protection, Chinese Academy of Agricultural Sciences, China) increased the attraction of *T. japonicum* and enhanced parasitism of *Chilo suppressalis* eggs. In contrast, Zhu et al. [57] reported that amino-oligosaccharide treatment reduced rice attractiveness to *T. japonicum* and decreased parasitism by 33.2%. The treatment altered 11 emitted volatiles, including methyl salicylate, decanal, D-limonene, 6-methyl-5-hepten-2-one, octanal, and eucalyptol, which showed concentration-dependent effects on parasitoid orientation. Metabolomic analysis also indicated increased salicylic-acid biosynthesis and trypsin-protease-inhibitor activity. These contrasting findings demonstrate that the effects of induced plant defense on parasitoid recruitment depend on the elicitor and the resulting volatile composition. Therefore, plant defense treatments should be evaluated for their combined effects on herbivore suppression, parasitoid attraction, and realized parasitism. Further comparisons across crop genotypes and field conditions are needed to identify defense strategies compatible with *Trichogramma*-based biological control.

### 2.6. Plant-Mediated Nutritional Effects on Host Egg Quality and Parasitoid Performance

Plant-mediated changes in herbivore nutrition can influence host egg quality and subsequent *Trichogramma* performance. Because immature parasitoids develop within host eggs, variation in egg size and nutritional composition may affect their development, survival, and reproduction. Sun et al. [58] investigated these effects by rearing Samia ricini on castor, Nepalese coriaria, or cassava, *Manihot esculenta* and subsequently evaluating the resulting eggs using six *Trichogramma* species. Silkworms feeding on castor developed faster, attained greater body and pupal mass, and produced wider eggs. For most parasitoid species, eggs from castor- or cassava-fed hosts supported higher parasitism and emergence than those from hosts feeding on *Nepalese coriaria*. However, responses varied among parasitoid species, and development was prolonged in several species using castor-derived eggs. These findings demonstrate that host-plant identity can influence parasitoid performance through changes in herbivore condition and egg characteristics.

Nevertheless, plant-mediated nutritional effects are not always transmitted to parasitoids. Costa et al. [59] reported that the Manteigado common-bean genotype reduced *Chrysodeixis includens* larval survival by 27% and contained approximately 21% less total protein than the comparison cultivar. However, neither plant genotype nor water availability significantly affected the measured biological parameters of *T. pretiosum* developing in the resulting eggs. In contrast, Sharma et al. [60] found that *Corcyra cephalonica* reared on sorghum exhibited faster development and greater fecundity, while *T. chilonis* and *T. achaeae* developing from sorghum-reared host eggs showed shorter developmental and pre-oviposition periods and higher population growth parameters. Parasitoid fecundity did not differ significantly among host diets, indicating that nutritional effects may vary among fitness traits. Together, these studies show that host-plant and herbivore nutrition can influence egg suitability, although responses depend on the plant–herbivore–parasitoid combination. Future research should examine how plant nutritional conditions affect host egg composition and whether these changes predict parasitoid development, emergence, and reproductive performance.

### 2.7. Plant Architecture, Canopy Microclimate, and Trichogramma Dispersal

Plant architecture can influence *Trichogramma* dispersal and the spatial distribution of parasitism. Oliveira et al. [61] reported that the mean dispersal distance of *T. pretiosum* in sweet corn increased from 4.7 m at the V4 stage to 5.9 m at V8, while the estimated dispersal area increased from 48.6 to 60.3 m^2^. Mean dispersal distances were 6.0 m in cucumber and 5.6 m in cabbage, with parasitism declining as distance from the release point increased. Similarly, da Silva Ponce et al. [62] recorded mean dispersal distances of 6.26 m in kale and 6.07 m in cabbage, corresponding to dispersal areas of 81.23 and 79.03 m^2^, respectively. These findings indicate that crop structure and growth stage can influence parasitoid movement and the spatial coverage of parasitism.

Environmental conditions within crop canopies can also affect parasitoid activity. Grande et al. [63] reported increased parasitism by *T. pretiosum* under light conditions, indicating that illumination influences host-searching activity. Chen et al. [64] found that the proportion of plants containing eggs parasitized by *T. achaeae* increased from 56.25% at 15 °C and 68.75% at 20 °C to 90.62% at 25 °C and 88.67% at 30 °C. Females also showed greater parasitism in the upper plant tier, whereas leaf surface had no significant effect. These findings indicate that temperature and vertical position can influence parasitoid host searching and parasitism. Furthermore, Martel et al. [65] reported reduced emergence of *T. ostriniae* as parasitized eggs remained exposed for longer periods under maize-field conditions, highlighting the importance of field exposure for immature parasitoid survival.

## 3. Plant-Mediated Chemical Signaling and *Trichogramma* Ecological Responses

### 3.1. Plant Volatiles and Trichogramma Host-Searching Responses

**Figure 1:**
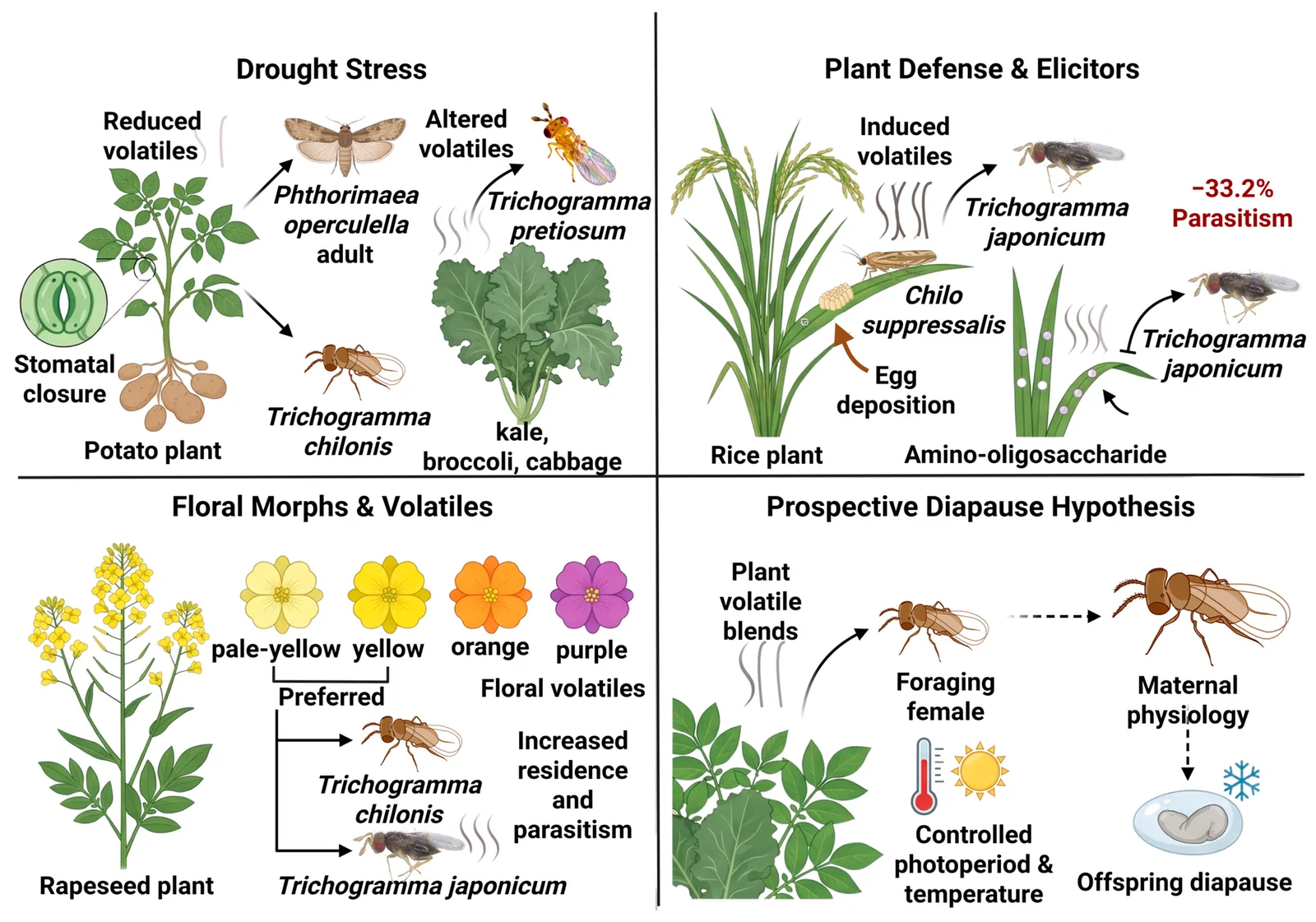
Plant-mediated effects on *Trichogramma* host-searching responses and their hypothesized implications for offspring diapause.

Plant volatiles provide important chemical cues that influence host-searching behavior in *Trichogramma*, with their effects depending on plant physiological conditions, herbivore activity, and volatile composition. He et al. [66] demonstrated that drought-induced stomatal closure reduced volatile emissions in potato plants, increasing their attractiveness to *Phthorimaea operculella* while reducing attraction of *T. chilonis* (Figure 1). In contrast, Ramalho et al. [67] reported that water stress altered volatile profiles in kale, broccoli, and cabbage, consistently increasing the attraction of *T. pretiosum* despite differences in herbivore oviposition responses (Figure 1). These contrasting findings indicate that drought effects on parasitoid host searching depend on the specific plant–herbivore–parasitoid system. Further evidence highlights the importance of plant defense responses and volatile composition. Yuan et al. [68] showed that egg deposition by *Chilo suppressalis* induced rice volatiles that attracted *T. japonicum* (Figure 1). Conversely, Zhu et al. [57] found that amino-oligosaccharide-induced changes in rice volatiles reduced *T. japonicum* attraction and parasitism by 33.2% (Figure 1). Bu et al. [69] further demonstrated that *T. chilonis* and *T. japonicum* preferred pale-yellow and yellow rapeseed flowers over orange or purple flowers, with differences in floral volatiles associated with improved parasitoid residence and parasitism performance (Figure 1). Collectively, these studies demonstrate that plant-derived chemical cues can either enhance or reduce parasitoid attraction and host-searching performance. However, whether plant volatiles influence maternal physiology and subsequent diapause induction in *Trichogramma* remains unknown. Although volatile-mediated changes in host searching and feeding opportunities could indirectly affect maternal reproductive condition, their consequences for offspring diapause have not been experimentally established. Future studies should therefore examine maternal exposure to different plant volatile blends under controlled temperature, photoperiod, and host-resource conditions, while measuring both behavioral responses and offspring diapause incidence (Figure 1).

### 3.2. Induced Plant Defenses and Trichogramma Recruitment

Induced plant defense responses can influence herbivore performance and *Trichogramma* host-searching behavior by altering plant volatile emissions. Yuan et al. [68] demonstrated that *Chilo suppressalis* egg deposition induced changes in rice volatiles, including D-limonene, linalool, nonanal, and methyl salicylate. Behavioral assays revealed dose-dependent attraction of female *T. japonicum* to specific compounds, indicating that rice plants can recruit egg parasitoids before herbivore larval feeding begins. Similarly, Murali-Baskaran et al. [70] reported that *Spodoptera litura* herbivory increased volatile diversity in pulse crops, with induced compounds such as octadecane and eicosane reducing herbivore feeding while enhancing *T. japonicum* foraging activity. Parasitism reached approximately 94–95% under the tested conditions. These findings demonstrate that induced plant defenses can simultaneously influence herbivore performance and parasitoid recruitment. However, whether defense-induced chemical changes also affect maternal physiology, host egg quality, and subsequent diapause induction in *Trichogramma* remains unknown. Future studies should integrate plant defense chemistry, host quality, and maternal responses with offspring diapause measurements under controlled photothermal conditions.

## 4. Environmental and Biological Regulation of *Trichogramma* Diapause

### 4.1. Diapause and Quiescence: Physiological and Developmental Distinctions

Diapause and quiescence both involve reduced or arrested development, but they differ fundamentally in their regulation. Diapause is a stage-specific developmental program that is initiated in anticipation of unfavorable conditions and involves coordinated induction, maintenance, and termination. Quiescence is a more immediate developmental response to adverse conditions and generally ends when permissive conditions return [71]. In *Trichogramma*, diapause commonly occurs at the prepupal stage within the parasitized host egg, although induction conditions vary among species [72,73]. Developmental stage is particularly important because sensitivity to diapause-inducing conditions can depend on the age of the developing parasitoid, as demonstrated in *T. dendrolimi* [74]. Therefore, studies investigating diapause should specify both the developmental stage at which diapause occurs and the timing of exposure to inducing conditions. This distinction is particularly important in *Trichogramma*, because low-temperature storage can produce prolonged developmental arrest without necessarily demonstrating that a diapause program has been activated. Recent synthesis of parasitoid diapause likewise emphasizes that diapause should be identified using developmental and physiological criteria rather than inferred from developmental delay alone [12]. Recent storage experiments provide useful empirical examples. Hasan et al. [75] exposed prepupal *T. evanescens* and *T. chilonis* to low-temperature storage following acclimation and interpreted the resulting developmental suppression primarily as quiescence. Storage for relatively short periods retained acceptable biological performance, whereas progressively prolonged exposure reduced emergence and subsequent productivity. The important point is not simply that development ceased at low temperature, but that developmental arrest was directly associated with exposure to the unfavorable condition and progressively accumulated physiological costs. Thus, cold-induced arrest alone is insufficient evidence of diapause.

Mashal et al. [76] more explicitly separated the two storage strategies in *T. evanescens*. Their experiments showed that an induction regime around 9 °C could support a long-duration diapause-based storage program extending for several months, whereas treatment around 11 °C was more suitable for shorter quiescent storage. Under an effective diapause-induction protocol, substantial adult emergence remained possible after storage extending to six months. This contrast indicates that successful long-term dormancy depends on preparatory physiological programming rather than simply more intense or prolonged refrigeration. Mechanistically, this distinction is consistent with the energetic organization of diapause. A diapausing parasitoid must enter developmental arrest with sufficient metabolic reserves and a physiological program capable of reducing expenditure while preserving the ability to resume development. Quiescence does not necessarily require the same anticipatory reorganization. Consequently, delayed emergence, low-temperature survival, or temporary developmental arrest should not be classified as diapause unless the study demonstrates an appropriate induction phase, characteristic developmental stage, persistence or regulation of arrest, and a recognizable termination response [8,12,77]. These criteria should also be applied when interpreting developmental delays under fluctuating field temperatures.

### 4.2. Temperature-Dependent Induction, Maintenance, and Termination of Diapause

Temperature is one of the strongest environmental regulators of *Trichogramma* diapause, but its effect cannot be represented by a simple relationship in which progressively lower temperatures produce progressively greater diapause incidence. Instead, diapause depends on the timing, magnitude, and duration of thermal exposure, together with the developmental stage and biological background of the parasitoid. Reznik and Voinovich [78] demonstrated a photoperiod-independent thermal response in *Trichogramma telengai* larvae, showing that temperature itself can contribute to diapause induction rather than operating solely as a modifier of photoperiod. The response did not follow a simple monotonic relationship with decreasing temperature. This is physiologically important because diapause induction requires the developing parasitoid to remain sufficiently functional to execute a regulated developmental transition. Moderately low temperature may therefore provide seasonal information while allowing diapause preparation, whereas thermal conditions outside the responsive range may suppress normal development without producing the same programmed state.

Temperature remains important after diapause has been established. Zhang et al. [79] showed that diapause termination could be accelerated by progressive temperature increases rather than a single constant low-temperature regime. Stepwise warming treatments improved diapause termination efficiency and subsequent biological performance, indicating that termination depends on the thermal trajectory rather than only the final temperature. Therefore, diapause induction, maintenance, and termination should be considered distinct thermal processes [8,12]. An inductive thermal window can initiate diapause, subsequent low temperatures can maintain developmental arrest, and an appropriate warming sequence can promote diapause completion and resumed development. These phases should not be interpreted simply as consequences of low-temperature exposure. Because field temperatures fluctuate across time and microhabitats, whereas laboratory studies commonly use constant regimes, experimental temperature thresholds should be interpreted cautiously. Future studies should incorporate realistic field temperature histories to determine how thermal fluctuations influence diapause induction, maintenance, and termination.

### 4.3. Photoperiod–Temperature Interactions in Maternal Diapause Regulation

Photoperiod provides a reliable seasonal signal, whereas temperature can modify diapause responses in *Trichogramma*. In *T. telengai*, Voinovich and Reznik [80] demonstrated that constant temperature and daily temperature fluctuations influence maternal photoperiodic responses, although thermoperiod cannot replace day length as an independent seasonal cue. Reznik and Voinovich [81] further showed that temperature affects two distinct stages of this response. Temperature during the scotophase influenced night-length measurement, showing an inverted U-shaped relationship, whereas temperature during the following photophase positively affected diapause induction in offspring. These findings indicate that photoperiod and temperature interact in regulating maternal diapause responses. Consequently, climatic warming may alter diapause induction even when seasonal day length remains unchanged. Alongside temperature and photoperiod, humidity may also influence diapause regulation in *Trichogramma*. Tang et al. [82] demonstrated that temperature and relative humidity significantly affected parasitism, development, and emergence in *T. dendrolimi* and *Trichogrammatoidea lutea*, with *T. dendrolimi* showing greater tolerance across different environmental conditions. Similarly, Cherif et al. [83] reported that temperature and humidity influenced several life-history traits in two *Trichogramma cacoeciae* strains. Emergence declined markedly at 35 °C, and the strains differed in their heat tolerance. These findings suggest that humidity, together with temperature, influences parasitoid development and survival. However, its direct contribution to diapause induction remains to be experimentally established.

### 4.4. Maternal and Transgenerational Effects on Diapause

Maternal and transgenerational effects can influence diapause regulation in *Trichogramma* across successive generations. Reznik et al. [84] demonstrated that lower grandmaternal temperatures increased winter-diapause incidence in *T. telengai* grandprogeny by approximately 20–25% under short-day conditions, suggesting temperature-dependent modification of the photoperiodic response. Reznik and Voinovich [85] further reported that diapause induction capacity in *Trichogramma principium* was reduced immediately after diapause but gradually recovered over 9–16 post-diapause generations, with higher temperatures accelerating recovery. These findings demonstrate that diapause responses can be influenced by environmental and physiological effects extending across generations.

### 4.5. Metabolic Regulation of Diapause in Trichogramma

Host-derived nutrients may influence diapause capacity in *Trichogramma* through lipid and carbohydrate metabolism. Zhang et al. [86] reported that silencing fatty acid synthase (FAS) in *T. dendrolimi* reduced diapause incidence by approximately 28% and negatively affected parasitism, ovarian development, and mature egg production. Similarly, Zhang et al. [87] demonstrated that silencing the trehalose transporters Tret1 and Tret1-2 impaired diapause expression and altered carbohydrate metabolism and hormone-related pathways. These findings highlight the importance of lipid biosynthesis and trehalose transport in diapause regulation. However, whether plant-mediated changes in host egg nutritional composition influence these metabolic pathways and subsequent diapause induction remains unclear. Host physiological condition may also influence the developmental environment experienced by immature *Trichogramma*. Variation in host egg age, nutritional composition, and physiological status can affect parasitoid development and performance. However, whether the diapause-related physiological state of the herbivore host directly modifies diapause induction in *Trichogramma* remains unclear. Host diapause and parasitoid diapause should therefore be treated as distinct biological processes rather than assumed to be synchronized. Future studies should compare host eggs differing in physiological or diapause-related status under standardized temperature and photoperiod conditions, while directly measuring parasitoid diapause incidence and subsequent development.

### 4.6. Geographic and Population-Level Variation in Diapause

Diapause induction in *Trichogramma* varies among geographic populations and individuals. Zhang et al. [88] demonstrated substantial geographic variation in *T. dendrolimi* under identical thermal treatments, with diapause incidence reaching 97.42% and 95.94% in Heilongjiang and Liaoning populations, respectively, compared with only 29.56% in the Jiangsu population. These differences suggest that populations may differ in their thermal sensitivity and diapause induction thresholds. Variation also occurs within populations. Similarly, Voinovich and Reznik [89] examined 428 isofemale substrains of *T. telengai* and found considerable differences in offspring diapause incidence, although these responses showed weak consistency across generations. This suggests that individual variation may involve physiological or environmental influences rather than stable inherited differences alone. Together, these studies indicate that diapause expression depends on population origin and individual environmental responses. However, whether geographic variation in diapause reflects local adaptation to crop phenology, seasonal host availability, and plant-mediated resource conditions remains unclear. Further studies integrating population origin, photothermal conditions, and host-resource quality are needed to clarify these relationships. Additional studies addressing the physiological, environmental, and biological-control aspects of *Trichogramma* diapause are summarized in Table 1.

## 5. Molecular and Physiological Mechanisms Underlying *Trichogramma* Diapause

Diapause is a regulated developmental program involving coordinated physiological and molecular adjustments that enable insects to survive predictable periods of environmental adversity. Unlike quiescence, diapause involves anticipatory developmental regulation accompanied by changes in energy metabolism, hormonal signaling, and stress tolerance. These processes are controlled by interconnected endocrine, metabolic, and transcriptional pathways that contribute to diapause induction, maintenance, and termination [105,106]. In *Trichogramma*, the molecular mechanisms underlying diapause remain incompletely understood. Zhang et al. [90] investigated diapause-associated gene expression in *T. dendrolimi* using comparative transcriptomic analysis and identified extensive differential expression of genes involved in signal transduction, transcriptional regulation, lipid and carbohydrate metabolism, oxidation–reduction processes, and cellular stress responses. These findings suggest that diapause involves coordinated molecular regulation, although transcriptomic associations alone cannot establish the causal functions of individual genes. More recently, Yang et al. [107] developed stage- and species-specific RNA interference (RNAi) approaches for *Trichogramma*, providing methodological opportunities to investigate candidate gene functions experimentally. Nevertheless, how environmental signals, maternal conditions, and host-derived nutritional resources interact with molecular pathways to influence diapause induction remains poorly understood. Future research should integrate functional gene analysis with controlled ecological experiments to determine how external cues are translated into physiological and molecular responses associated with diapause regulation.

### 5.1. Endocrine Regulation of Trichogramma Diapause

Endocrine signaling coordinates developmental transitions, metabolism, and diapause in insects through interactions among juvenile hormone (JH), ecdysteroids, insulin/insulin-like growth factor signaling (IIS), and target of rapamycin (TOR) pathways. These regulatory networks integrate developmental and nutritional signals to influence growth suppression, energy allocation, and stress tolerance [105,106]. However, direct evidence for their involvement in *Trichogramma* diapause remains limited. Transcriptomic analysis of *T. dendrolimi* identified differentially expressed genes associated with developmental regulation and signal transduction, including forkhead-box (FOX)-related transcription factors [90]. In other insect systems, forkhead box O (FOXO), a downstream component of IIS, contributes to metabolic regulation and stress resistance, while IIS/TOR signaling influences the balance between growth and energy conservation [106]. Nevertheless, the involvement of a specific IIS–FOXO–TOR regulatory pathway in *Trichogramma* diapause has not been functionally established. Future research should combine measurements of JH and ecdysteroids with analyses of IIS/TOR activity and functional manipulation of candidate genes, including FOXO, to determine their roles in diapause induction, maintenance, and termination. Such investigations would help establish whether endocrine mechanisms identified in other insects are conserved in *Trichogramma* and how they respond to environmental and nutritional cues.

### 5.2. Lipid Metabolism and Energy Allocation During Diapause

Lipid metabolism plays an important role in insect diapause by supporting energy storage, metabolic maintenance, and recovery following developmental arrest [8,25]. In *Trichogramma*, transcriptomic analysis of *T. dendrolimi* identified differential expression of genes associated with fatty acid synthesis, lipid transport, and energy metabolism between diapause and non-diapause individuals [90]. More direct evidence was provided by Zhang et al. [86], who demonstrated that RNA interference-mediated silencing of fatty acid synthase (FAS), a key enzyme in fatty acid biosynthesis, reduced diapause induction by approximately 28% in *T. dendrolimi*. FAS silencing also impaired parasitism, ovarian development, and mature egg production without significantly affecting female longevity. Changes in the expression of reproduction-associated genes, including those related to vitellogenin signaling, further suggested that fatty acid metabolism contributes to both diapause-associated physiology and reproductive development [86]. These findings provide functional evidence linking FAS activity with diapause incidence, although they do not establish whether altered lipid reserves directly mediate the observed response. More broadly, lipid metabolism supports reproductive investment and energy allocation in insects [108], suggesting a potential physiological connection between diapause preparation and reproduction. However, the relative contributions of lipid synthesis, storage, and mobilization during different diapause stages remain unclear. Future studies should combine quantitative lipid profiling, functional gene manipulation, and measurements of host-derived nutritional resources to determine how lipid availability influences diapause induction, maintenance, and post-diapause performance.

### 5.3. Wolbachia and Symbiont-Associated Variation in Diapause

Endosymbiotic microorganisms can influence insect physiology, reproduction, and environmental responses, potentially modifying diapause-related traits [109,110]. Among these microorganisms, *Wolbachia* is particularly important in *Trichogramma* because certain infections induce thelytokous parthenogenesis, allowing females to produce daughters without fertilization [111,112,113]. Rahimi-Kaldeh et al. [114] investigated diapause and circadian clock gene expression in sexual and *Wolbachia*-associated asexual strains of *Trichogramma brassicae*. Photoperiod influenced diapause induction in both strains, whereas the asexual strain exhibited lower diapause incidence and reduced expression of several clock-associated genes, including clk, cyc, per, timeout, and cry2. These findings suggest an association among reproductive mode, circadian regulation, and diapause responsiveness. However, because infected and uninfected strains may also differ genetically, these comparisons alone cannot establish that *Wolbachia* directly causes the observed differences.

Pintureau et al. [115] further investigated interactions between *Wolbachia* infection and diapause in *Trichogramma*, reporting that infection-related effects varied among the reproductive and developmental traits examined. Their findings also suggested that diapause conditions could influence the expression of *Wolbachia*-associated reproductive characteristics. Together, these studies indicate that symbiont–diapause relationships may be context dependent rather than universally positive or negative. Although broader insect research suggests possible links between microbial symbionts, host nutrition, and stress tolerance [110,116], the underlying mechanisms in *Trichogramma* remain insufficiently characterized. Future investigations should compare genetically matched infected and cured lines, quantify symbiont density, and integrate clock-gene expression, metabolic profiling, and diapause measurements under standardized environmental conditions. These approaches would help distinguish direct symbiont effects from host genetic and environmental influences.

## 6. Climate Change, Diapause Regulation, and Seasonal Synchrony in *Trichogramma*

Climate change poses a complex challenge to parasitoid-based biological control because it alters not only mean temperature but also the temporal relationships among temperature, photoperiod, plant development, herbivore phenology, drought, and extreme events [28,117,118]. In *Trichogramma*, diapause induction is influenced by temperature, photoperiod, maternal conditions, and population-specific responses [81,84,85,88,92,119,120,121,122,123]. Because photoperiod remains astronomically stable while seasonal temperature patterns change, warming may alter the correspondence between diapause-inducing cues and actual environmental conditions. Climate-related changes in plant phenology, host availability, and volatile-mediated interactions may further affect parasitoid performance and seasonal synchrony [28,118,124,125,126]. However, the combined consequences of these environmental changes for *Trichogramma* diapause and biological-control effectiveness remain insufficiently investigated.

### 6.1. Warming and Changes in Diapause Induction

Temperature influences diapause induction in *Trichogramma* through species- and population-dependent responses rather than a uniform relationship with cooling. Geographic variation in diapause induction has been documented in *T. dendrolimi* [88], while studies of *T. telengai* demonstrate that thermal conditions can contribute to diapause induction independently of photoperiod and modify maternal photoperiodic responses [78,81]. Ma and Chen [74] further demonstrated that diapause induction in *T. dendrolimi* depended on temperature, exposure duration, and developmental age. Diapause was induced at 7–10 °C following approximately 4–6 weeks of exposure, with the highest sensitivity occurring at early developmental stages. These findings indicate that successful diapause induction requires appropriate combinations of thermal conditions and developmental timing. Consequently, climate warming may alter the frequency, duration, and seasonal timing of temperature conditions suitable for diapause induction. However, the consequences for diapause incidence and parasitoid–host synchrony remain uncertain because responses depend on species, population origin, developmental stage, and interactions between temperature and photoperiod. Future experiments should evaluate fluctuating temperature regimes alongside maternal photoperiod and host-resource availability to determine how warming influences diapause incidence and timing across geographic populations.

### 6.2. Spring Warming, Diapause Termination, and Phenological Mismatch

Spring warming may influence parasitoid–host synchrony by altering diapause termination and subsequent adult emergence. Zhang et al. [79] demonstrated that gradually increasing temperature shortened diapause termination in *T. dendrolimi* compared with conventional constant-temperature storage. Temperature treatments involving sequential increases also improved parasitism and emergence rates, indicating potential benefits for laboratory production. However, these controlled-storage results do not establish that natural spring warming necessarily causes premature field emergence. Earlier emergence could instead create a phenological mismatch if suitable host eggs are unavailable when parasitoids become active.

Evidence from other parasitoid systems illustrates the importance of host phenology. Gould et al. [127] examined the introduced parasitoid *Tetrastichus planipennisi* in relation to its invasive emerald ash borer host, highlighting the potential influence of host developmental timing and climatic conditions on parasitoid establishment. However, this introduced biological-control system differs from native parasitoid–host associations because the interacting species have not necessarily undergone long-term local coadaptation with host trees and climatic conditions. Consequently, its findings should be interpreted as context-specific evidence rather than directly extrapolated to native systems or *Trichogramma*. Further research is needed to determine how host phenology, local adaptation, and climate variability jointly influence parasitoid establishment and seasonal synchrony.

### 6.3. Extreme Temperature Events and Diapause Disruption

Extreme temperature events may affect *Trichogramma* diapause differently from gradual seasonal temperature changes. Voinovich and Reznik [128] demonstrated that brief cold (−10 °C) and heat (43 °C) shocks lasting at least 20–30 min reduced diapause incidence in T. telengai larvae. However, the underlying responses differed: heat shocks primarily altered the relative representation of diapause-destined and non-diapause-destined individuals, whereas cold-shock effects were largely associated with differential mortality. Sensitivity also depended on developmental stage, highlighting the importance of exposure timing [128]. These findings indicate that reduced diapause incidence following thermal stress should not automatically be interpreted as direct inhibition of diapause programming. Temperature-dependent changes in parasitism have also been documented in *T. achaeae* microcosms [64]. At a broader scale, Salcido et al. [129] analyzed 22 years of plant–caterpillar–parasitoid interactions in a protected tropical forest and reported declines in insect diversity, abundance, and parasitism, partly associated with climatic changes and weather anomalies. Although these ecological findings do not establish effects on *Trichogramma* diapause, they illustrate the potential consequences of climatic variability for parasitoid-mediated ecosystem services. Future research should distinguish gradual warming, daily temperature fluctuations, and acute thermal shocks by measuring diapause incidence, stage-specific survival, emergence timing, and post-diapause reproductive performance under realistic climatic scenarios.

## 7. Conclusions

Plants influence *Trichogramma* ecology and biological-control performance by affecting host egg availability, volatile-mediated host searching, host quality, and environmental conditions. The studies reviewed here demonstrate that these effects vary among plant species, herbivores, parasitoid species, and environmental conditions. Temperature, photoperiod, maternal effects, and metabolic processes are established components of *Trichogramma* diapause regulation, whereas the direct contribution of plant-derived cues remains poorly understood. Although plant-mediated changes in host availability, nutrition, and microclimate may influence seasonal adaptation, their effects on diapause induction require experimental confirmation. Future studies should examine these relationships by combining controlled plant and host treatments with measurements of maternal responses, offspring diapause, metabolic regulation, and post-diapause performance. Climate change may further alter seasonal synchrony by modifying temperature regimes, crop development, and host availability. Integrating plant ecology with parasitoid physiology and field-based research will help clarify the mechanisms linking plant-associated conditions to diapause and support the development of more reliable *Trichogramma*-based biological control strategies.

## Figures and Tables

**Table 1:** Additional evidence on diapause regulation, physiological responses, and related biological-control traits in *Trichogramma*.

Study	*Trichogramma* Species/System	Factor Examined	Principal Evidence	Mechanistic or Conceptual Relevance	Evidence Status
Zhang et al. [90]	*Trichogramma dendrolimi*	Diapause transcriptomics	High-throughput profiling identified extensive differential gene expression between diapause and non-diapause developmental states.	Supports coordinated transcriptional reprogramming during diapause rather than simple developmental slowing.	Direct diapause evidence
Xu et al. [91]	*T. ostriniae*	Diapause exposure across generations	Diapause-exposed parasitoids showed enhanced parasitism and longevity through F0–F2, with traits returning toward non-diapause levels by F3.	Indicates that diapause-associated effects on reproductive performance and longevity can persist across subsequent generations.	Direct diapause evidence
Reznik and Voinovich [92]	*T. telengai*	Maternal response to a single long night	A detectable increase in offspring diapause occurred within hours; diapause incidence subsequently increased to high levels over approximately 30–50 h.	Shows unusually rapid conversion of maternal photoperiodic information into offspring developmental programming.	Direct diapause evidence
Ghosh and Ballal [93]	*T. chilonis*	Maternal thermophotoperiod	Maternal temperature–photoperiod conditioning enhanced diapause induction in a laboratory line and supported prolonged storage.	Provides applied evidence that maternal history can determine offspring diapause competence.	Direct diapause evidence
Ghosh and Ballal [94]	*T. chilonis* strains	Thermal induction and termination	Several strains entered prepupal diapause under defined prestorage thermal and photoperiodic treatment, whereas the conventional laboratory strain responded differently.	Demonstrates strain dependence of induction and the need for population-specific diapause protocols.	Direct diapause evidence
Cagnotti et al. [95]	*Trichogramma nerudai*	Acclimation before cold storage	A longer acclimation period substantially improved tolerance of extended cold storage and preserved several post-storage quality traits.	Demonstrates that pre-storage acclimation improves cold-storage tolerance, although this does not establish diapause induction.	Dormancy/storage support
Haque et al. [96]	*T. evanescens*	Host species, host age and cold storage	Storage response depended on host species, host egg age and storage duration, with corresponding changes in parasitism and emergence.	Demonstrates interaction between host condition and cold-storage performance.	Host/storage support
Du et al. [97]	*T. dendrolimi* and *T. chilonis* produced through multiparasitism	Duration of cold storage	Biological characteristics changed with progressively longer storage, with responses depending on parasitoid species and production context.	Reinforces that storage tolerance is phenotype- and species-dependent rather than a uniform property of the genus.	Dormancy/storage support
Zhang et al. [98]	*T. dendrolimi*	Diapause versus conventional cold storage	Diapaused parasitoids generally retained stronger production and parasitism performance than conventionally cold-stored individuals across generations.	Demonstrates practical biological advantages of programmed diapause over direct cold storage.	Direct diapause evidence
Xu et al. [99]	*T. dendrolimi*	Adult supplementary sugars	Sugar supplementation improved longevity and parasitism performance in mass-rearing conditions.	Establishes that adult energetic state can strongly affect reproductive performance, relevant to testing maternal energetic pathways to diapause.	Indirect nutritional evidence
Yuan et al. [100]	*T. chilonis* on *Samia cynthia ricini* eggs	Fertilization, collection method and host egg storage	Unfertilized naturally laid eggs supported stronger rearing performance, whereas excessive host egg or parasitized-egg storage reduced reproductive output.	Demonstrates how host condition and storage history determine the resource environment encountered by developing parasitoids.	Indirect host-quality evidence
Zhang et al. [101]	*T. dendrolimi* *T. chilonis* *Trichogramma leucaniae* *T. ostriniae* *T. japonicum* and *T. pretiosum*	Eri-silkworm egg preparation	Unfertilized and washed eri-silkworm eggs generally provided high suitability for mass production across several parasitoid species.	Shows that physical and physiological host egg state can substantially alter parasitoid performance.	Indirect host-quality evidence
Xue et al. [102]	*T. leucaniae*	Factitious-host identity	Eri-silkworm eggs supported high and stable parasitism/emergence during multigenerational rearing.	Supports host-resource quality as a major determinant of sustained parasitoid production and physiological condition.	Indirect host-quality evidence
Zhang et al. [103]	*Trichogramma* spp. on *Corcyra cephalonica*	Vacuum packaging and host egg cold storage	Vacuum packaging delayed host egg protein degradation and improved suitability during an approximately 20-day storage window.	Provides a biochemical mechanism linking host-storage treatment, protein preservation and subsequent parasitoid performance.	Indirect resource evidence
Tabebordbar et al. [104]	Three populations of *Trichogramma euproctidis*	Population origin and host age	Life-table performance differed among parasitoid populations and with host age.	Demonstrates population- and host-dependent variation in life-history performance, providing indirect context for investigating diapause differences among populations.	Indirect population evidence

## Data Availability

No new empirical dataset was generated for this review. Data sharing is not applicable.
